# Pakistan Randomized and Observational Trial to Evaluate Coronavirus Treatment (PROTECT) of Hydroxychloroquine, Oseltamivir and Azithromycin to treat newly diagnosed patients with COVID-19 infection who have no comorbidities like diabetes mellitus: A structured summary of a study protocol for a randomized controlled trial

**DOI:** 10.1186/s13063-020-04616-4

**Published:** 2020-08-08

**Authors:** Javed Akram, Shehnoor Azhar, Muhammad Shahzad, Waqas Latif, Khalid Saeed Khan

**Affiliations:** 1 Vice Chancellor/Professor of Internal Medicine, University of Health Sciences, Lahore, Pakistan; 2 Assistant Professor Public Health, University of Health Sciences, Lahore, Pakistan; 3 Professor of Pharmacology, University of Health Sciences, Lahore, Pakistan; 4 Data Analyst at University of Health Sciences, Lahore, Pakistan; 5 Distinguished Investigator at Department of Preventive Medicine and Public Health, University of Granada, Granada, Spain

**Keywords:** COVID-19, Randomised controlled trial, protocol, SARS-CoV-2, Hydroxychloroquine, Azithromycin, Oseltamivir, multi-center, adaptive, randomization

## Abstract

**Objectives:**

To evaluate the effectiveness of Hydroxychloroquine Phosphate/Sulfate (200 mg orally 8 hourly thrice a day for 5 days), *versus* oseltamivir (75 mg orally twice a day for 5 days), and *versus* Azithromycin (500 mg orally daily on day 1, followed by 250 mg orally twice a day on days 2-5) alone and in combination (in all seven groups), in clearing the coronavirus (COVID-19) nucleic acid from throat and nasal swab and in bringing about clinical improvement on day 7 of follow-up (primary outcomes).

**Trial Design:**

An adaptive design, set within a comprehensive cohort study, to permit flexibility in this fast-changing clinical and public health scenario. The randomized study will be a multicenter, multiarm, multistage, randomized controlled trial with a parallel design. An observation only cohort will emerge from those not consenting to randomization.

**Participants:**

Eligible will be newly diagnosed patients, either hospitalized or in self-isolation, without any comorbidities or with controlled chronic medical conditions like diabetes mellitus and hypertension. Participants of any gender or age group having tested positive for COVID-19 on Real-Time qRT-PCR (Quantitative Reverse Transcription PCR) will be invited to take part in study at twelve centers across eight cities in Pakistan. Those pregnant or lactating, severely dyspneic or with respiratory distress, already undergoing treatment, and with serious comorbidities like liver or kidney failure will be excluded.

**Intervention and Comparator:**

There will be a total of seven comparator groups: Each drug (Hydroxychloroquine Phosphate/Sulfate, Oseltamivir and Azithromycin) given as monotherapy (three groups); combinations of each of two drugs (three groups); and a final group on triple drug regimen.

**Main Outcomes:**

The laboratory-based primary outcome will be turning the test negative for COVID-19 on qRT-PCR on day 7 of follow-up. The clinical primary outcome will be improvement from baseline of two points on a seven-category ordinal scale of clinical status on day 7 of follow-up.

**Randomization:**

Participants will be randomized, maintaining concealment of allocation sequence, using a computer-generated random number list of variable block size into multiple intervention groups in the allocation ratio of 1:1 for all groups.

**Blinding (masking):**

This is an open label study, neither physician nor participants will be blinded.

**Numbers to be randomized (sample size):**

This is an adaptive design and parameters for formal sample size calculation in a new disease of a previously unknown virus are not available. Thus, the final sample size will be subjected to periodic reviews at each stage of adaptive design and subsequent advice of National Data Safety & Management Board (NDSMB) notified by Drug Regulatory Authority of Pakistan.

**Trial Status:**

Protocol Version 1.7 dated July 5, 2020. By July 03, 2020, the trial had recruited a total of about 470 participants across 12 centers after approval from the National Bioethics Committee and Drug Regulatory Authority of Pakistan. Recruitment started on April 20, 2020. The recruitment is expected to continue for at least three months subject to review by the National Data Safety and Management Board (NDSMB) notified by Drug Regulatory Authority of Pakistan.

**Trial Registration:**

Prospectively registered on 8 April 2020 at **clinicaltrials.gov ID:** **NCT04338698**

*The full protocol is attached as an additional file, accessible from the Trials website (Additional file* *1**). In the interest in expediting dissemination of this material, the familiar formatting has been eliminated; this Letter serves as a summary of the key elements of the full protocol.*

*The study protocol has been reported in accordance with the Standard Protocol Items: Recommendations for Clinical Interventional Trials (SPIRIT) guidelines (Additional file* *2**).*

## Supplementary information

**Additional file 1.** Full Study Protocol.

**Additional file 2.** SPIRIT 2013 Checklist: Recommended items to address in a clinical trial protocol and related documents.

## Data Availability

NDSMB will be the custodian of the final trial data and investigators will give an undertaking for not using it in part of whole for any purpose without prior written authorization from the NDSMB. Subject to NDSMB written approval, any part or whole of the protocol, site-specific data, or the entire dataset could be made available to the public for academic use only.

