# Correction to: Pakistan Randomized and Observational Trial to Evaluate Coronavirus Treatment (PROTECT) of Hydroxychloroquine, Oseltamivir and Azithromycin to treat newly diagnosed patients with COVID-19 infection: A structured summary of a study protocol for a randomized controlled trial

**DOI:** 10.1186/s13063-020-04697-1

**Published:** 2020-08-22

**Authors:** Javed Akram, Shehnoor Azhar, Muhammad Shahzad, Waqas Latif, Khalid Saeed Khan

**Affiliations:** 1 Vice Chancellor/Professor of Internal Medicine, University of Health Sciences, Lahore, Pakistan; 2 Assistant Professor Public Health, University of Health Sciences, Lahore, Pakistan; 3 Professor of Pharmacology, University of Health Sciences, Lahore, Pakistan; 4 Data Analyst at University of Health Sciences, Lahore, Pakistan; 5 Distinguished Investigator at Department of Preventive Medicine and Public Health, University of Granada, Granada, Spain

**Correction to: Trials 21, 702 (2020)**

**https://doi.org/10.1186/s13063-020-04616-4**

Following publication of the original article [1], we were notified that the authors would like to modify the article title to “Pakistan Randomized and Observational Trial to Evaluate Coronavirus Treatment (PROTECT) of Hydroxychloroquine, Oseltamivir and Azithromycin to treat newly diagnosed patients with COVID-19 infection: A structured summary of a study protocol for a randomized controlled trial”, as it is conceptually and grammatically more accurate and reflective of the nature and topic.
