# Supplementary material for: Pakistan Randomized and Observational Trial to Evaluate Coronavirus Treatment (PROTECT) of Hydroxychloroquine, Oseltamivir and Azithromycin to treat newly diagnosed patients with COVID-19 infection who have no comorbidities like diabetes mellitus: A structured summary of a study protocol for a randomized controlled trial
Source: Trials. 2020 Aug 8;21:702. doi: 10.1186/s13063-020-04616-4 (PMC7414257; doi:10.1186/s13063-020-04616-4)
Supplement: Supplementary file 1 — Additional file 1. Full Study Protocol. [file 13063_2020_4616_MOESM1_ESM.docx]

**Pakistan Randomized and Observational Trial to Evaluate Coronavirus Treatment (PROTECT): A Structured Summary**

***Hydroxychloroquine, Oseltamivir and Azithromycin for the treatment of COVID-19 infection: A Randomized Controlled Clinical Trial***

**ClinicalTrials.gov ID: NCT04338698**

**Protocol Version: 1.7 date July 3, 2020**

[**theprotect.com.pk**](theprotect.com.pk)

All funding and coordination was arranged by the University of Health Sciences (UHS) Lahore, Pakistan with technical assistance from Federal Task Force on COVID-19

**Authors:**

- Javed Akram, Professor or of Internal Medicine and Vice Chancellor UHS (jakram@gmail.com)
- Shehnoor Azhar, Assistant Professor Public Health UHS **(Corresponding author:** shehnoor.azhar@gmail.com)
- Waqas Latif, Data Analyst UHS (waqas341@gmail.com))
- Muhammad Shahzad, Professor of Pharmacology Department of Pharmacology UHS (shahzad912@gmail.com)
- Khalid Saeed Khan, Professor of Preventive Medicine and Public Health, University of Granada Spain (profkkhan@gmail.com)

**Abstract**

Objectives

To evaluate the effectiveness of Hydroxychloroquine Phosphate/Sulfate (200 mg orally 8 hourly thrice a day for 5 days) *versus* oseltamivir (75 mg orally twice a day for 5 days) *versus* Azithromycin (500 mg orally daily on day 1, followed by 250 mg orally twice a day on days 2-5) alone and in combination (in all seven groups), in clearing the coronavirus (COVID-19) nucleic acid from throat and nasal swab and in bringing about clinical improvement on day 7 of follow-up (primary outcomes).

Trial Design

An adaptive design, set within a comprehensive cohort study, is chosen because it permits flexibility in this fast-changing clinical and public health scenario. The randomized study will be a multicenter, multiarm, multistage, randomized controlled trial with a parallel design. An observation only cohort will emerge from those not consenting to randomization.

Participants

Eligible will be newly diagnosed patients, either hospitalized or in self-isolation, without any comorbidities or with controlled chronic medical conditions like diabetes mellitus and hypertension. Participants of any gender or age group having tested positive for COVID-19 on Real-Time qRT-PCR (Quantitative Reverse Transcription PCR) will be invited to take part in study at twelve centers across nine cities in Pakistan. Those pregnant or lactating, severely dyspneic or under respiratory distress, already taking a treatment, and with serious co-morbidities like liver or kidney failure will be excluded.

Intervention and Comparator

A total of seven comparator groups will be formed: Each drug (Hydroxychloroquine Phosphate/Sulfate, Oseltamivir and Azithromycin) given as monotherapy (three groups); combinations of each of two drugs (three groups); and a final group on triple drug regimen.

Main Outcomes

The laboratory-based primary outcome will be turning test negative for COVID-19 on qRT-PCR on day 7 of follow-up. The clinical primary outcome will be improvement from baseline of two points on a seven-category ordinal scale on day 7 of follow-up.

Randomization

Participants will be randomized, maintaining concealment of allocation sequence, using a computer-generated random number list of variable block size into multiple intervention groups.

Blinding

Computerized allocation to treatment group will be done upon adding participants demographic details and confirmation of informed consent. Neither physician nor participants will be blinded.

Sample Size

This is an adaptive design and parameters for formal sample size calculation in a new disease of a previously unknown virus are not available. Then the sample size and power analysis are indicative for review at each stage of adaptive design. In a standard two-arm, head-to-head trial, the sample size would have been as follows: in order to detect a difference in the laboratory-based primary outcome of 10% (assuming a 50% of participants receiving Hydroxychloroquine Phosphate/Sulfate turn test negative at day 7 and expected rates in comparator groups of 60%), at a significance level 5% and a power of 90%, a sample size of approximately 520 subjects in each group will be required. This sample size will be enough to detect a small-medium difference (Cohen’s d 0.3, a significance level 5% and a power of 90%) in the clinical primary outcome.

Trial Status

By July 03, 2020, the trial had recruited a total of about 470 participants across 12 centers after approval from National Bioethics Committee and Drug Regulatory Authority of Pakistan. Recruitment started on April 20, 2020. The recruitment is expected to continue for at least next three months subject to review of data monitoring and safety board as estimated in Version 1.7.

Trial Registration

Prospectively registered at **clinicaltrials.gov ID: NCT04338698**

*The full protocol is attached as an additional file, accessible from the Trials website (Final_1.7_Plain)*

*The study protocol has been reported in accordance with the Standard Protocol Items: Recommendations for Clinical Interventional Trials (SPIRIT) guidelines and this is a PDF version of protocol 1.7 without citations for specific sections nested at the top (SPIRIT_Nested,pdf)*

Key Words

SARS-CoV-2, Hydroxychloroquine, Azithromycin, Oseltamivir, multi-center, adaptive, randomization

Declarations

Authors declare no financial or any competing interests

Ethics Approval

The proposed study is approved by National Bioethics Committee of Pakistan **(notification attached)** and IRBs at majority of participating sites. Remaining sites have endorsed the ethics approval of both University of Health Sciences Lahore and National Bioethics Committee (NBC).

Certified that this trial has received ethical approval from the appropriate ethical committee as described above

Consent for publication

Applicable through this submission

Availability of data and materials

NDSMB will be the custodian of the final trial data and investigators will give an undertaking for not using it in part of whole for any purpose without prior written authorization from the NDSMB.

Subjected to NDSMB written approval, any part or whole of the protocol, site-specific data, or the entire dataset could be made available to public for academic use only.

Competing interests

The authors declare that they have no competing interests

Funding

All Funding arranged by UHS Lahore

Authors' contributions

- JA, KSK, SA, WL wrote the Hypothesis or Comparator groups, Primary and Secondary Outcomes
- SA wrote Introduction, part of Methods and Ethical Approvals, adapted manuscript to SPIRIT Guidelines
- KSK, SA, WL wrote sections on Eligibility Criteria and Data Safety & Monitoring
- MS devised Dosage and Informed Consent Tool
- WL contributed with Sample Size Calculations, Data Analysis

Acknowledgements

- Authors acknowledge continuous support of Federal Task Force on Covid-19 under its Chairman Prof Atta ur Rahman.
- Also thanking laboratory personnel and faculty at UHS in supporting this effort
- External Peer reviewer Prof Javier Zamora, Head of Clinical Biostatistics Unit, Hospital Ramon y Cajal, IRYCIS, Madrid Spain
- Professor Khan is Distinguished Investigator at University of Granada funded by the Beatriz Galindo (senior modality) program of the Spanish Ministry of Education.

***SPIRIT Guidance: Background and rationale***

*6a Description of research question and justification for undertaking the trial, including summary of relevant studies (published and unpublished) examining benefits and harms for each intervention*

**Introduction**

Initial warnings that all countries would suffer SARS-Cov-2 (Covid-19) pandemic in 2020 have come true. With mean incubation period reported around 6 to 7 days, the disease is characterized by fever, sore throat, dyspnea, loss of smell and taste that could progress to irreversible damage to lungs leading to death. The global fatality rate of 3 – 4% has been estimated. In the continued absence of a definitive treatment or vaccine, this pandemic continues to harm public health, lifestyles, and economies around the world.

Hydroxychloroquine Sulphate (HQ), commonly used against malaria and rheumatoid arthritis in Indo-Asian region, was reported to stop symptomatic progression of SARS-Cov-2 by inhibiting pH dependent steps of viral replication. It’s effectiveness as monotherapy or in combination with common antibiotics and antivirals is being evaluated around the world. Non-randomized study of Guatret et al promoted the use of macrolides like Azithromycin with HQ in clearing the virus at day 6 among hospitalized patients. None of the 21 patients enrolled in this study reported any adverse drug reactions. Recruitment in HQ arm of ongoing Solidarity Trial resumed after initial concerns of cardiotoxicity were addresses by data safety review. To date, several countries have seen widespread compassionate use of HQ and Azithromycin during the ongoing pandemic.

*6b Explanation for choice of comparators*

Ulrich and Pillat have attributed interest in common antibiotic Azithromycin (Macrolide) to its mechanism of action against rhinovirus - interference with ligand/CD147 receptor interactions and decrease expression of some metalloproteinases (downstream to CD147) that inhibit viral replication. Oseltamivir was one of the earliest drugs tested on SARS-CoV-2 patients in Wuhan, China. With proven effectiveness against influenza viruses, it was evaluated as monotherapy and in combinations to rule out sharing of the same neuraminidase enzyme by novel Coronavirus.

***SPIRIT Guidance:*** *Objectives*

*7 Specific objectives or hypotheses*

**Objectives**

***Primary Objective***

- To evaluate the effectiveness of Hydroxychloroquine Phosphate/Sulfate (200 mg orally 8hr thrice a day for 5 days) versus oseltamivir (75 mg orally twice a day for 5 days) versus Azithromycin (500 mg orally daily on day 1, followed by 250 mg orally twice a day on days 2-5) as monotherapy and in various combinations in clearing the coronavirus nucleic acid from throat and nasal swab and in bringing about clinical improvement on day 7 of follow-up (primary outcomes).

***2.1 Secondary Objectives***

- To evaluate various clinical parameters, quality of life, and symptoms scores, within all randomized participants stratified by subgroups
- To follow up participants not consenting to randomization for the outcomes of SARS-Cov-2 infection with supportive treatment only

**3 STUDY DESIGN**

***SPIRIT Guidance: Study Design***

*8 Description of trial design including type of trial (eg, parallel group, crossover, factorial, single group), allocation ratio, and framework (eg, superiority, equivalence, noninferiority, exploratory)*

An adaptive design, set within a comprehensive cohort study, is chosen because it permits flexibility in this fast-changing clinical and public health scenario. The randomized study will be a multicenter, multiarm, multistage, randomized controlled trial with a parallel design.

***Methods: Participants, interventions, and outcomes***

**4 PARTICIPANTS**

***SPIRIT Guidance: Study setting***

*9 Description of study settings (eg, community clinic, academic hospital) and list of countries where data will be collected. Reference to where list of study sites can be obtained*

Study sites comprise major public-sector tertiary care health facilities and Covid-19 field hospitals being setup within province of Punjab. Hospitals in other provinces and federal areas will also be invited for participation. Households with self-isolating individuals could also participate in the study. A list of hospitals with confirmed participation has been attached as **Annex 3.**

***SPIRIT Guidance:*** *Eligibility criteria*

*10 Inclusion and exclusion criteria for participants. If applicable, eligibility criteria for study centers and individuals who will perform the interventions (eg, surgeons, psychotherapists)*

Eligible will be newly diagnosed patients without any comorbidities or those with controlled chronic medical conditions, e.g. diabetes mellitus and hypertension. Participants of either gender or age group having tested positive for COVID-19 on qRT-PCR (Quantitative Reverse Transcription PCR) will be invited to take part. Participants who are pregnant or lactating, are already taking any treatment, under respiratory distress or severely dyspneic, have liver and kidney failure will be excluded from the study. Each participant will undergo baseline investigation i.e., liver function tests, renal function tests, urinalysis, and Complete Blood Count; at the time of enrollment. However, diagnostic facilities and hospital admission criteria could vary across cities. A case report form (CRF) has been developed for this proposal and **attached** at the end of this document.

***Inclusion Criteria***

1. Confirmed SARS-CoV-2 (COVID-19) infection by a positive test result
2. Either gender
3. Symptomatic for example fever, dry cough, myalgias

***Exclusion Criteria***

1. Confirmed absence of SARS-CoV-2 (COVID-19) infection by a negative test result
2. Have chronic conditions such as heart disease, liver and kidney failure
3. Ongoing respiratory distress or severely dyspneic
4. Pregnant or currently lactating female
5. Immunocompromised, atopic and/or systemic disease(s)
6. On other antiviral drugs
7. History of allergy to any of the drugs to be administered in this study

Both recruitment and administration of study drugs will be carried out by any member of the site investigation team. All site teams have been trained accordingly.

***SPIRIT Guidance:*** *Interventions*

*11a Interventions for each group with enough detail to allow replication, including how and when they will be administered*

Participants will be divided into seven intervention groups (A to G) and eighth group (H) comprising non-consenting individuals observed for supportive care (Figure 1). Study physician(s) will administer three drugs as monotherapy (to three intervention arms), in combination regimen of any of the two trial drugs (to three additional arms), and seventh intervention arm comprising all three drugs. The three drugs with respective dosages are - Hydroxychloroquine Phosphate/Sulfate (200 mg orally 8hr thrice a day for 5 days) versus oseltamivir (75 mg orally twice a day for 5 days) versus Azithromycin (500 mg orally daily on day 1, followed by 250 mg orally twice a day on days 2-5)

***SPIRIT Guidance:***

*11b Criteria for discontinuing or modifying allocated interventions for a given trial participant (eg, drug dose change in response to harms, participant request, or improving/worsening disease)*

Study physicians will observe, interview, and report any suspected adverse drug reaction (sADR) to their unit teams and subsequently to electronic data entry module. “A sADR is an unwanted or harmful reaction experienced following the administration of a drug or combination of drugs under normal conditions of use and is suspected to be related to the drug”. In case of sADR, the participant will be informed of discontinuation of given therapy until resolution of symptoms is observed and/or receipt of corresponding investigations (Electrocardiogram, Hepatotoxicity, Nephrotoxicity, Myelotoxicity) confirm the suspicion or otherwise. Findings will be conveyed to participant and recorded on electronic data module. A written report of any confirmed episode will also be immediately submitted to Drug Regulatory Authority of Pakistan (DRAP) on its prescribed proforma **(attached** as sample). Any decision to modify the study protocol or drug dosage will be informed by aggregated data on ADRs and Drug Regulatory Authority is a statutory forum to exercise these decisions (if warranted) through pre-notified National Data Safety & Monitoring Board (NDSMB). Irrespective of sADR, the study participants reserve the right to withdraw their informed consent at any point of time throughout recruitment period.

***SPIRIT Guidance:***

*11c Strategies to improve adherence to intervention protocols, and any procedures for monitoring adherence (eg, drug tablet return, laboratory tests)*

Administration of drugs to participants will be directly observed by the clinical staff at the study sites. Each of the site team has been trained to maintain a record of pharmaceutical supplies from stock entries to disposition. All supplies are labelled NOT FOR SALE from the source. The site teams will be responsible to project demand corresponding with the rate of recruitment, monitor appropriate usage, and maintain a folder of used drug packaging for site audits.

***SPIRIT Guidance:***

*11d Relevant concomitant care and interventions that are permitted or prohibited during the trial*

The site physicians are responsible to exercise their clinical judgement in provision of best diagnostic and therapeutic care to enrolled patients beyond this protocol, and record each/any of those within patient file and electronic data entry module.

***SPIRIT Guidance: Outcomes***

*12 Primary, secondary, and other outcomes, including the specific measurement variable (eg, systolic blood pressure), analysis metric (eg, change from baseline, final value, time to event), method of aggregation (eg, median, proportion), and time point for each outcome. Explanation of the clinical relevance of chosen efficacy and harm outcomes is strongly recommended*

Investigators will ensure collection of nasopharyngeal and oropharyngeal swab samples from participants at Days 7 and 14 (those positive at day 7 will be followed up further till day 14 or beyond) for monitoring of primary endpoint - measuring time to turn test negative for COVID-19 on qRT-PCR (Quantitative Reverse Transcription PCR). Swab samples will be taken by a trained ENT (ear-nose-throat) nurse as per local guidelines and sent to laboratory as per biosafety protocol **(attached)**. The clinical progression or recession in symptoms would be monitored and reported on the 7-point ordinal scale on each of days 3, 5, 7, 9, 11, and 14. For hospitalized participants, baseline investigations such as CBC, Urea electrolytes, kidney (Creatinine), liver (Liver Function Tests), and cardiac functions (Enzymes, D-dimers, ECG) are to be recorded preferably at the time of admission to hospital and as frequently as permissible within given resources. All participants should have undergone these investigations at least once during the recruiting period. Diagnostics such as Electrocardiogram, Hepatotoxicity, Nephrotoxicity, Myelotoxicity are indicated immediately upon suspected ADR as explained on Page 8 (Methods). It has to be mentioned that clinical and diagnostic facilities for Covid-19 vary across study sites due to administrative and geographical factors known to shape emergency responses.

Secondary outcomes like Quality of Life and Symptoms Score will be recorded on day 0 and 7 using standardized tools such as World Health Organization Quality of Life Questionnaire (WHO QoL) and Wisconsin Upper Respiratory Symptoms Survey (WURSS) respectively (copies **attached)**. The former is expected to capture participant’s lived experience during care that has not been documented in literature to date. The latter is expected to indicate any respiratory co-infection including those acquired from the hospital. The investigators will also monitor and record variables including duration it takes to oxygenate and ventilate, duration stayed oxygenated and ventilate, number of hospitals stay days, admission to intensive care, and mortality. This methodology has been peer reviewed by an external expert **(see annex 1).** A GCP certified, experienced researcher will conduct the training of up to 50 investigators across 13 participating sites to prepare them for all aspects of this study.

***SPIRIT Guidance: Participant timeline***

*13 Time schedules of enrolment, interventions (including any run-ins and washouts), assessments, and visits for participants. A schematic diagram is highly recommended (see Figure)*

Flow Chart **(Figure 1)**

***SPIRIT Guidance: Sample size***

*14 Estimated number of participants needed to achieve study objectives and how it was determined, including clinical and statistical assumptions supporting any sample size calculations*

This is an adaptive design and parameters for formal sample size calculation in a new disease of a previously unknown virus are not available. Then the sample size and power analysis are indicative for review at each stage of adaptive design. In a standard two-arm, head-to-head trial, the sample size would have been as follows: in order to detect a difference in the laboratory-based primary outcome of 10% (assuming a 50% of participants receiving Hydroxychloroquine Phosphate/Sulfate turn test negative at day 7 and expected rates in comparator groups of 60%), at a significance level 5% and a power of 90%, a sample size of approximately 520 subjects in each group will be required. This sample size will be enough to detect a small-medium difference (Cohen’s d 0.3, a significance level 5% and a power of 90%) in the clinical primary outcome. Additional considerations will need to make for the interim analysis at every stage of the adaptive design. Interim analysis will review sample size according to time to clearance also. Planned blinded sample size re-estimations will need to be undertaken in which we will constantly re-examine the original sample size assumptions. We will aim to maintain the trial power at 90% even if the original sample size assumptions turn out to be far from the observed data. The adaptation at each stage will allow us to drop out fewer promising interventions and will prevent an underpowered trial for the most promising interventions. As the planned modifications will be undertaken in a blinded fashion, there will be no increase in the type I error rate.

***SPIRIT Guidance: Recruitment***

*15 Strategies for achieving adequate participant enrolment to reach target sample size*

All patients fulfilling the eligibility criteria and providing informed consent are eligible for this study. Going by the projections, it is expected to enroll substantial number of participants from clinical facilities. A coordination group has been created on digital platform for immediate response to queries, learning from each other’s experiences, and continuity of operations. However, if health system indicates creep, investigators could recruit patients self-isolating at homes. Upon identification by site investigators in the hospital, this cohort would be contacted by trained researchers and data collectors via phone and if informed consent is obtained, recruited. The allocated treatment medicines for 14 days would be delivered. Similarly, swab samples and other clinical information would be collected from participants’ homes in case they cannot travel for testing. All other clinical data would be collected via phone and entered directly into the IT module. The unused or used drug packaging will be collected from participants’ homes upon completion of recruitment period of testing negative on PCR whichever comes first. It is expected that a wider pool of both hospitalized and home-isolated patients would be enough to meet the estimated sample size within the given timeframe.

**Methods: Assignment of interventions (for controlled trials)**

**Allocation:**

***SPIRIT Guidance: Sequence generation***

*16a Method of generating the allocation sequence (eg, computer-generated random numbers), and list of any factors for stratification. To reduce predictability of a random sequence, details of any planned restriction (eg, blocking) should be provided in a separate document that is unavailable to those who enroll participants or assign interventions*.

Participants will be randomized, maintaining concealment of allocation sequence, using a computer-generated random number list of variable block size into multiple intervention groups (and an observation only group will emerge from those not consenting to randomization). Stratification for age will be used initially to ensure that groups remain balanced in size and prognosis.

***SPIRIT Guidance: Allocation concealment mechanism***

*16b Mechanism of implementing the allocation sequence (eg, central telephone; sequentially numbered, opaque, sealed envelopes), describing any steps to conceal the sequence until interventions are assigned*

***SPIRIT Guidance: Implementation***

*16c Who will generate the allocation sequence, who will enrol participants, and who will assign participants to interventions*

Randomization sequence was created using Sealed Envelope Ltd 2019 and was stratified by study center and age using random block sizes of 28 and 42 sizes into seven treatments (11). Before randomizing, recruiting physician will confirm eligibility and obtain written informed consent from the participant. Upon entering participant’s demographic details in the IT module, a unique patient ID for the said center and treatment arm would be allocated automatically meaning the recruiting physician has no control over assigning intervention.

***SPIRIT Guidance: Blinding (masking)***

*17a Who will be blinded after assignment to interventions (eg, trial participants, care providers, outcome assessors, data analysts), and how*

*17b If blinded, circumstances under which unblinding is permissible, and procedure for revealing a participant’s allocated intervention during the trial*

Not Applicable

**Methods: Data collection, management, and analysis**

***SPIRIT Guidance: Data collection methods***

*18a Plans for assessment and collection of outcome, baseline, and other trial data, including any related processes to promote data quality (eg, duplicate measurements, training of assessors) and a description of study instruments (eg, questionnaires, laboratory tests) along with their reliability and validity, if known. Reference to where data collection forms can be found, if not in the protocol*

*18b Plans to promote participant retention and complete follow-up, including list of any outcome data to be collected for participants who discontinue or deviate from intervention protocols*

**6 DATA COLLECTION, MANAGEMENT AND ANALYSIS**

The COVID-19 PATIENTS RESEARCH PORTAL (IT Module) is an electronic case reporting form (CRF) developed by IT Department of University of Health Sciences, Lahore and accessible via <theprotect.com.pk>. It is expected to fully facilitate both investigators as well study participants. Using a computer desktop or any portable device such as personal computers and tablets, it allows input of data directly from patients’ bedsides for facilities that are electronically equipped and offer reliable internet within isolation areas. It was finalized after pilot testing at six study centers. Considering the highly contagious nature of this disease, the electronic CRF is expected to minimize cross-infection possible with use of printed study tools. The IT module has the option for investigators to withdraw a case should a participant wishes to discontinue enrollment in the study at any point of time.

***SPIRIT Guidance: Data management***

*19 Plans for data entry, coding, security, and storage, including any related processes to promote data quality (eg, double data entry; range checks for data values). Reference to where details of data management procedures can be found, if not in the protocol*

The role of IT Manager has been assigned to any member of the site team or his/her nominee. (S)he will have exclusive access to the data entry module under given terms of use to ensure data protection and privacy. Data will be entered immediately upon receipt. Sites are trained to develop a housekeeping system to keep track of local data including date stamping all documents on receipt. For data completion and validation, consistency checks would be performed during entry and warning(s) (if any) will be displayed. Data will be copied on a different server regularly. In case of an erroneous entry, the IT Manager will contact designated IT resource at UHS or Lead Biostatistician, provide the justification and get the issue resolved. Each of such correction request would be recorded.

***SPIRIT Guidance: Statistical methods***

*20a Statistical methods for analyzing primary and secondary outcomes. Reference to where other details of the statistical analysis plan can be found, if not in the protocol*

*20b Methods for any additional analyses (eg, subgroup and adjusted analyses)*

*20c Definition of analysis population relating to protocol non-adherence (eg, as randomized analysis), and any statistical methods to handle missing data (eg, multiple imputation)*

All trial data will be analyzed by biostatistician based at the University of Health Sciences, Lahore, using an a priori, approved analysis plan (SPSS 25.0). Interim analyses at each stage (first stage will be 10% of the total sample size) of the adaptive design will be pre-planned and undertaken confidentially keeping investigators and participants blind till the end of the trial. Primary and secondary outcomes will be analyzed in accordance to the group in which the participants were randomized deploying the intention-to-treat principle. The primary analysis will be expressed as odds ratio (OR) of the comparison between groups for the primary outcome. Interim analyses will be conducted without disclosing groups, thereby maintaining a level of blinding in interpretation of interim results. The confidential interim analyses at each stage of the adaptive design will examine for imbalance in co-morbidity at baseline. If any differences are observed then stratification may be modified confidentially. The results will be reported as point estimates and 95% confidence intervals. Secondary analysis will include in time-to-event data to directly compare the data among groups with survival plots and hazard ratios. This will permit evaluation of the decision to continue beyond Day 7 of treatment upon testing negative, potential drug effects besides informing valuable understanding of drug effectiveness. Stratified analyses will be done by baseline COVID-19 positive status upon testing and age groups. Multiple regression and Cox proportional hazards stratified analysis will be used to adjust for a small number of covariates taken from stratification factors if there were baseline imbalances. Secondary analyses will deploy multiple regression models as well Chi square, Fisher’s exact test and one-way ANOVA to compare groups. The two-sided p-value less than or equal to 0.05 will be taken as significant. Imputation method will be used to handle missing data.

**Methods: Monitoring**

***SPIRIT Guidance: Data monitoring***

*21a Composition of data monitoring committee (DMC); summary of its role and reporting structure; statement of whether it is independent from the sponsor and competing interests; and reference to where further details about its charter can be found, if not in the protocol. Alternatively, an explanation of why a DMC is not needed*

**8 DATA SAFETY AND MONITORING**

The independent National Data Safety and Monitoring Board (NDSMB) has been notified by Drug Regulatory Authority of Pakistan to oversee all Covid-19 research **(Annex II).** It comprises eminent physicians, researchers, biostatisticians and officials that will scrutinize data for compliance with given patient safety standards as per Good Clinical Practices. It’s also vested with authority to decide on halting a study or an intervention arm until a subsequent review is conducted. It is supervised by the Chairman and each of the meeting is recorded by the designated Secretary.

***SPIRIT Guidance: Data monitoring***

*21b Description of any interim analyses and stopping guidelines, including who will have access to these interim results and make the final decision to terminate the trial*

In line with well-established editorial standards, the interim analyses will be done independently of the authors of the paper and investigators. To achieve that, NDSMB will establish an interim analysis sub-committee of independent individuals who will be named in acknowledgment section of the paper. The study statistician will be part of this committee and will give a written undertaking to keep that interim findings blind from the clinical investigators. The NDSMB meeting agenda will have an open and a closed part, the latter part will only by attended by interim analysis sub-committee. All decisions including termination of the study and related observations will be communicated to the investigators in writing by the Secretary of NDSMB.

***SPIRIT Guidance: Harms***

*22 Plans for collecting, assessing, reporting, and managing solicited and spontaneously reported adverse events and other unintended effects of trial interventions or trial conduct*

Diagnostics such as Electrocardiogram, Hepatotoxicity, Nephrotoxicity, Myelotoxicity are indicated once ADR is suspected and explained on Page 8 (Methods). Any additional incident or finding defined as adverse event (AE) during recruitment period will be reported to IT module. An adverse event (AE) is “any untoward medical occurrence in a patient or clinical investigation subject administered a pharmaceutical product that does NOT necessarily have a causal relationship with this treatment. For example, a physical injury resulting from fall or slip, a natural calamity during in-patient stay etc”. Investigators are also trained to ensure safe and secure environment for participants to provide necessary psychosocial support, when indicated.

***SPIRIT Guidance: Auditing***

*23 Frequency and procedures for auditing trial conduct, if any, and whether the process will be independent from investigators and the sponsor*

The NDSMB can convene under its Chairman at anytime to independently review and audit findings and provide subsequent guidance on remaining conduct of the study. Its composition ensures that any conflicts of interests are avoided and patient safety and protection are prioritized.

**Ethics and dissemination**

***SPIRIT Guidance: Research ethics approval***

*24 Plans for seeking research ethics committee/institutional review board (REC/IRB) approval*

*Protocol amendments*

The proposed study is approved by National Bioethics Committee of Pakistan (notification **attached)** and IRBs at majority of participating sites. Remaining sites have endorsed the ethics approval of both University of Health Sciences Lahore and National Bioethics Committee (NBC).

*25 Plans for communicating important protocol modifications (eg, changes to eligibility criteria, outcomes, analyses) to relevant parties (eg, investigators, REC/IRBs, trial participants, trial registries, journals, regulators)*

The approval from NBC is binding on primary investigators to keep it informed of any modifications in protocol should they arise in view of any of the factors including but not limited to emerging science or knowledge of disease that was previously unknown, clinical feedback or from participating patient(s), or subsequent to NDSMB review. Subsequent to any approval of protocol modifications, lead investigators will be responsible for re-training or other changes to be communicated to each of the site.

*Consent or assent 26a Who will obtain informed consent or assent from potential trial participants or authorized surrogates, and how (see Item 32)*

No participant could be randomized without obtaining informed consent. It is the responsibility of the investigator to clearly communicate to potential participants the study objectives and lack of any definitive treatment of SARS-Cov-2. All investigators are trained GCP guidelines have been incorporated in the training module on informed consent so that investigators encourage potential participants to seek any clarification regarding study drugs, expected benefits and harms. In case written informed consent was not obtained due to clinical practices that bar use of paper in isolation area or obtained via phone, the circumstances and justification has to be mentioned in case reports Contact details of all patients must be maintained as a separate dataset for auditors to verify the compliance with protocol. A printed version of informed consent form in local language has been developed and delivered to each site in enough numbers. For isolation areas allowing the use of printed copies, maintaining a separate folder for informed consent of all enrollees has been advised.

*26b Additional consent provisions for collection and use of participant data and biological specimens in ancillary studies, if applicable*

No biological specimen will be stored for any ancillary studies.

*Confidentiality 27 How personal information about potential and enrolled participants will be collected, shared, and maintained in order to protect confidentiality before, during, and after the trial*

All printed data (informed consent, CRF, diagnostic reports, death certificates) will be maintained in individual case files. All printed data will be secured in a separate storage area under strict custody of the site lead researcher. The electronic record of enrollment will identify enrollees as unique identification numbers generated at the time of randomization. All participants will be informed that the study data will be maintained for the period of one year and will be destroyed afterwards in all forms – electronic and paper.

***SPIRIT Guidance: Declaration of interests***

*28 Financial and other competing interests for principal investigators for the overall trial and each study site*

None

***SPIRIT Guidance: Access to data***

*29 Statement of who will have access to the final trial dataset, and disclosure of contractual agreements that limit such access for investigators*

NDSMB will be the custodian of the final trial data and investigators will give an undertaking for not using it in part of whole for any purpose without prior written authorization from the NDSMB.

***SPIRIT Guidance: Ancillary and post-trial care***

30 Provisions, if any, for ancillary and post-trial care, and for compensation to those who suffer harm from trial participation

The study is conducted in view of lack of treatment options for mild to moderately symptomatic SARS-Cov-2 with a commitment to no-harm practices and a robust adverse drug reaction monitoring framework.

***SPIRIT Guidance: Dissemination policy***

*31a Plans for investigators and sponsor to communicate trial results to participants, healthcare professionals, the public, and other relevant groups (eg, via publication, reporting in results databases, or other data sharing arrangements), including any publication restrictions*

Subsequent to NDSMB review and approval, findings of interim analysis and relevant results are to be announced publicly and submitted for publication in a scientific journal for dissemination to the scientific community and public at large represented by the study participants.

*31b Authorship eligibility guidelines and any intended use of professional writers*

The final manuscript would be published as group authorship with investigators’ names mentioned in acknowledgement section in alphabetical order.

*31c Plans, if any, for granting public access to the full protocol, participant level dataset, and statistical code*

If NDSMB permits, any part or whole of the protocol, site-specific data, or the entire dataset could be made available to public for academic use only.
