# Supplementary material for: Pakistan Randomized and Observational Trial to Evaluate Coronavirus Treatment (PROTECT) of Hydroxychloroquine, Oseltamivir and Azithromycin to treat newly diagnosed patients with COVID-19 infection who have no comorbidities like diabetes mellitus: A structured summary of a study protocol for a randomized controlled trial
Source: Trials. 2020 Aug 8;21:702. doi: 10.1186/s13063-020-04616-4 (PMC7414257; doi:10.1186/s13063-020-04616-4)
Supplement: Supplementary file 2 — Additional file 2. SPIRIT 2013 Checklist: Recommended items to address in a clinical trial protocol and related documents. [file 13063_2020_4616_MOESM2_ESM.doc]

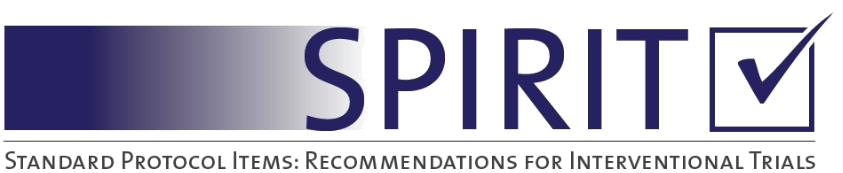


SPIRIT 2013 Checklist: Recommended items to address in a clinical trial protocol and related documents*

| **Section/item** | **Item** | **Description** |
| --- | --- | --- |
|  | **No** |  |
|  | | |
| **Administrative information** | | |
| Title | 1 | Descriptive title identifying the study design, population, interventions, |
|  |  | and, if applicable, trial acronym  Structured summary page 1 |
| Trial registration | 2a | Trial identifier and registry name. If not yet registered, name of |
|  |  | intended registry  Structured summary page 1 |
|  | 2b | All items from the World Health Organization Trial Registration Data |
|  |  | Set |
| Protocol version | 3 | Date and version identifier  Structured summary page 1 |
| Funding | 4 | Sources and types of financial, material, and other support  Structured summary page 4 |
| Roles and | 5a | Names, affiliations, and roles of protocol contributors  Structured summary page 5 |
| responsibilities | 5b | Name and contact information for the trial sponsor (List of authors on page 1) |
|  |
|  | 5c | Role of study sponsor and funders, if any, in study design; collection, |
|  |  | management, analysis, and interpretation of data; writing of the report; |
|  |  | and the decision to submit the report for publication, including whether |
|  |  | they will have ultimate authority over any of these activities  Structured summary page 4 |
|  | 5d | Composition, roles, and responsibilities of the coordinating centre, |
|  |  | steering committee, endpoint adjudication committee, data |
|  |  | management team, and other individuals or groups overseeing the |
|  |  | trial, if applicable (see Item 21a for data monitoring committee)  Structured summary page 4 and 5 |
| **Introduction** |  |  |
| Background and | 6a | Description of research question and justification for undertaking the |
| rationale |  | trial, including summary of relevant studies (published and |
|  |  | unpublished) examining benefits and harms for each intervention  Manuscript Page 1 |
|  | 6b | Explanation for choice of comparators Manuscript Page 1 |
| Objectives | 7 | Specific objectives or hypotheses Manuscript Page 2 |
| Trial design | 8 | Description of trial design including type of trial (eg, parallel group, |
|  |  | crossover, factorial, single group), allocation ratio, and framework (eg, |
|  |  | superiority, equivalence, noninferiority, exploratory) Manuscript Page 2 |

**Methods: Participants, interventions, and outcomes**

| Study setting | 9 | Description of study settings (eg, community clinic, academic hospital) |
| --- | --- | --- |
|  |  | and list of countries where data will be collected. Reference to where |
|  |  | list of study sites can be obtained Manuscript Page 2 |
| Eligibility criteria | 10 | Inclusion and exclusion criteria for participants. If applicable, eligibility |
|  |  | criteria for study centres and individuals who will perform the |
|  |  | interventions (eg, surgeons, psychotherapists) Manuscript Page 3 |
| Interventions | 11a | Interventions for each group with sufficient detail to allow replication, |
|  |  | including how and when they will be administered Manuscript Page 4 |
|  | 11b | Criteria for discontinuing or modifying allocated interventions for a |
|  |  | given trial participant (eg, drug dose change in response to harms, |
|  |  | participant request, or improving/worsening disease) Manuscript Page 4 |
|  | 11c | Strategies to improve adherence to intervention protocols, and any |
|  |  | procedures for monitoring adherence (eg, drug tablet return, |
|  |  | laboratory tests) Manuscript Page 5 |
|  | 11d | Relevant concomitant care and interventions that are permitted or |
|  |  | prohibited during the trial Manuscript Page 5 |
| Outcomes | 12 | Primary, secondary, and other outcomes, including the specific |
|  |  | measurement variable (eg, systolic blood pressure), analysis metric |
|  |  | (eg, change from baseline, final value, time to event), method of |
|  |  | aggregation (eg, median, proportion), and time point for each |
|  |  | outcome. Explanation of the clinical relevance of chosen efficacy and |
|  |  | harm outcomes is strongly recommended Manuscript Pages 5 and 6 |
| Participant | 13 | Time schedule of enrolment, interventions (including any run-ins and |
| timeline |  | washouts), assessments, and visits for participants. A schematic |
|  |  | diagram is highly recommended (see Figure 1 Page 15) |
| Sample size | 14 | Estimated number of participants needed to achieve study objectives |
|  |  | and how it was determined, including clinical and statistical |
|  |  | assumptions supporting any sample size calculations Manuscript Page 6 and 7 |
| Recruitment | 15 | Strategies for achieving adequate participant enrolment to reach |
|  |  | target sample size Manuscript Page 7 |

**Methods: Assignment of interventions (for controlled trials)**

Allocation:

Sequence 16a Method of generating the allocation sequence (eg, computer-

generation generated random numbers), and list of any factors for stratification.

To reduce predictability of a random sequence, details of any planned restriction (eg, blocking) should be provided in a separate document that is unavailable to those who enrol participants or assign interventions Manuscript Page 8

2

| Allocation | 16b | Mechanism of implementing the allocation sequence (eg, central |
| --- | --- | --- |
| concealment |  | telephone; sequentially numbered, opaque, sealed envelopes), |
| mechanism |  | describing any steps to conceal the sequence until interventions are |
|  |  | Assigned Manuscript Page 8 |
| Implementation | 16c | Who will generate the allocation sequence, who will enrol participants, |
|  |  | and who will assign participants to interventions Manuscript Page 8 |
| Blinding | 17a | Who will be blinded after assignment to interventions (eg, trial |
| (masking) |  | participants, care providers, outcome assessors, data analysts), and |
|  |  | How (N/A) |
|  | 17b | If blinded, circumstances under which unblinding is permissible, and |
|  |  | procedure for revealing a participant’s allocated intervention during |
|  |  | the trial (N/A) |

**Methods: Data collection, management, and analysis**

| Data collection | 18a | Plans for assessment and collection of outcome, baseline, and other |
| --- | --- | --- |
| methods |  | trial data, including any related processes to promote data quality (eg, |
|  |  | duplicate measurements, training of assessors) and a description of |
|  |  | study instruments (eg, questionnaires, laboratory tests) along with |
|  |  | their reliability and validity, if known. Reference to where data |
|  |  | collection forms can be found, if not in the protocol Manuscript Pages 5 and 6 |
|  | 18b | Plans to promote participant retention and complete follow-up, |
|  |  | including list of any outcome data to be collected for participants who |
|  |  | discontinue or deviate from intervention protocols Manuscript Pages 4 and 5 (attention to transparency in sharing any sADR) and pages 7 and 8 through recruitment even if patient is home-isolated. Informed consent in local language to clearly outline risks and benefits so that trust is developed between physician and participant |
| Data | 19 | Plans for data entry, coding, security, and storage, including any |
| management |  | related processes to promote data quality (eg, double data entry; |
|  |  | range checks for data values). Reference to where details of data |
|  |  | management procedures can be found, if not in the protocol Manuscript Pages 9, 10, 11 |
| Statistical | 20a | Statistical methods for analysing primary and secondary outcomes. |
| methods |  | Reference to where other details of the statistical analysis plan can be |
|  |  | found, if not in the protocol Manuscript Page 9, 10 |
|  | 20b | Methods for any additional analyses (eg, subgroup and adjusted |
|  |  | analyses) Manuscript Page 10 |
|  | 20c | Definition of analysis population relating to protocol non-adherence |
|  |  | (eg, as randomised analysis), and any statistical methods to handle |
|  |  | missing data (eg, multiple imputation) Manuscript Page 10 |
| **Methods: Monitoring** | |  |
| Data monitoring | 21a | Composition of data monitoring committee (DMC); summary of its role |
|  |  | and reporting structure; statement of whether it is independent from |
|  |  | the sponsor and competing interests; and reference to where further |

details about its charter can be found, if not in the protocol.

Alternatively, an explanation of why a DMC is not needed

Manuscript Page 10, 11, 26

| 3 | 21b | Description of any interim analyses and stopping guidelines, including |
| --- | --- | --- |
|  |  | who will have access to these interim results and make the final |
|  |  | decision to terminate the trial Manuscript Page 10 |
| Harms | 22 | Plans for collecting, assessing, reporting, and managing solicited and |
|  |  | spontaneously reported adverse events and other unintended effects |
|  |  | of trial interventions or trial conduct Manuscript Page 6, 7, 10 |
| Auditing | 23 | Frequency and procedures for auditing trial conduct, if any, and |
|  |  | whether the process will be independent from investigators and the |
|  |  | Sponsor Manuscript Page 11 |
| **Ethics and dissemination** | | |
| Research ethics | 24 | Plans for seeking research ethics committee/institutional review board |
| approval |  | (REC/IRB) approval Manuscript Page 12 |
| Protocol | 25 | Plans for communicating important protocol modifications (eg, |
| amendments |  | changes to eligibility criteria, outcomes, analyses) to relevant parties |
|  |  | (eg, investigators, REC/IRBs, trial participants, trial registries, journals, |
|  |  | regulators) Manuscript Page 12 |
| Consent or assent | 26a | Who will obtain informed consent or assent from potential trial |
|  |  | participants or authorised surrogates, and how (see Item 32)  Manuscript Page 12 |
|  | 26b | Additional consent provisions for collection and use of participant data |
|  |  | and biological specimens in ancillary studies, if applicable Manuscript Page 13 |
| Confidentiality | 27 | How personal information about potential and enrolled participants will |
|  |  | be collected, shared, and maintained in order to protect confidentiality |
|  |  | before, during, and after the trial Manuscript Page 13 |
| Declaration of | 28 | Financial and other competing interests for principal investigators for |
| interests |  | the overall trial and each study site Structured Summary Page 4 |
| Access to data | 29 | Statement of who will have access to the final trial dataset, and |
|  |  | disclosure of contractual agreements that limit such access for |
|  |  | Investigators Manuscript Page 13 |
| Ancillary and | 30 | Provisions, if any, for ancillary and post-trial care, and for |
| post-trial care |  | compensation to those who suffer harm from trial participation  Manuscript Page 13 |
| Dissemination | 31a | Plans for investigators and sponsor to communicate trial results to |
| policy |  | participants, healthcare professionals, the public, and other relevant |
|  |  | groups (eg, via publication, reporting in results databases, or other |
|  |  | data sharing arrangements), including any publication restrictions  Manuscript Page 13 |
|  | 31b | Authorship eligibility guidelines and any intended use of professional |
|  |  | Writers Manuscript Page 14 |
|  | 31c | Plans, if any, for granting public access to the full protocol, participant- |
|  |  | level dataset, and statistical code Manuscript Page 14 |

| **Appendices** |  |  |
| --- | --- | --- |
| Informed consent | 32 | Model consent form and other related documentation given to |
| materials |  | participants and authorised surrogates (additional files attached to email) |
| Biological | 33 | Plans for collection, laboratory evaluation, and storage of biological |
| specimens |  | specimens for genetic or molecular analysis in the current trial and for |
|  |  | future use in ancillary studies, if applicable N/A |

*It is strongly recommended that this checklist be read in conjunction with the SPIRIT 2013 Explanation & Elaboration for important clarification on the items. Amendments to the protocol should be tracked and dated. The SPIRIT checklist is copyrighted by the SPIRIT Group under the Creative Commons [“Attribution-NonCommercial-NoDerivs 3.0 Unported](http://www.creativecommons.org/licenses/by-nc-nd/3.0/)” license.

5
